# Everyday Digital Technology Use and Youth Health: Scoping Review of Longitudinal Studies

**DOI:** 10.2196/85094

**Published:** 2026-04-27

**Authors:** Preetika Banerjee, Louise Holly

**Affiliations:** 1 Department of Pharmacy, CHOICE Institute, School of Pharmacy, University of Washington, Health Sciences Building, 1956 NE Pacific St H362, Seattle, WA, 98195, United States; 2 Digital Transformations for Health Lab, University of Geneva, Geneva, Switzerland

**Keywords:** digital technologies, digital health interventions, youth health, adolescent health, longitudinal studies, scoping review

## Abstract

**Background:**

Everyday digital technologies such as social media, gaming, and internet use are deeply integrated into the lives of children, adolescents, and young adults. While these platforms can foster connection, learning, and entertainment, concerns have grown about their potential to influence mental, physical, and social well-being. Research on this topic has expanded rapidly over the past decade, yet much of it remains cross-sectional, limiting insights into long-term outcomes. Longitudinal studies are essential to capture evolving patterns of digital engagement, identify causal relationships, and guide effective policies and interventions that support youth in navigating digital environments. In particular, evidence is needed to distinguish between beneficial and harmful forms of digital engagement, such as social connection versus problematic use, and to understand how these impacts differ across diverse populations and contexts. The COVID-19 pandemic further accelerated young people’s technology use, underscoring the urgency of examining both risks and opportunities. This review, therefore, synthesizes longitudinal research to map trends, identify knowledge gaps, and inform future directions.

**Objective:**

The study aimed to systematically identify and map longitudinal studies examining associations between everyday digital technology use (eg, social media, gaming, and internet use) and the health and well-being of youth (25 years or younger) and to chart the types of evidence available by technology category, outcomes, and geographical setting in order to highlight key gaps for future research.

**Methods:**

A systematic search of PubMed, Embase, and PsycArticles (2014‐2024) was conducted and reported in accordance with PRISMA-ScR (Preferred Reporting Items for Systematic Reviews and Meta-Analyses Extension for Scoping Reviews). Data extraction covered demographics, digital technology categories, and health outcomes. Studies were grouped into 6 key themes: social media use and mental health, digital addiction and behavioral outcomes, physical activity and digital technology, digital health technologies and cognitive development, parental influence and digital technology, and digital well-being and risk behaviors.

**Results:**

Of the 456 studies identified, 267 were longitudinal studies relevant to our research aims. Internet use (n=201 studies), social media (n=140 studies), and gaming (n=83 studies) dominated the themes. Mental health was the most frequently assessed outcome, with a focus on anxiety and depression. Geographically, 15% (40/267) of studies originated from low- and middle-income countries, with the majority from high-income settings such as the United States (n=76 studies) and Australia (n=15 studies). Nearly half (131/267, 49%) were published post 2020, reflecting heightened interest during the COVID-19 pandemic.

**Conclusions:**

Longitudinal evidence on everyday digital technology use and youth health is growing but remains concentrated in mental health outcomes and high-income settings, with notable gaps in physical health, educational outcomes, and equity-focused research. These findings highlight the need for more diverse, methodologically robust longitudinal studies to inform context-sensitive policies and interventions that balance the risks and benefits of digital engagement for young people.

## Introduction

### Digital Technology Use and Youth Health

The rapid expansion of digital technologies has profoundly shaped young people’s lives, introducing both opportunities and risks for their health and well-being [1]. Over the past decade, everyday digital technologies such as smartphones, social media platforms, and online games have become nearly ubiquitous in the lives of children and adolescents, with many young people spending several hours per day online across multiple devices [2]. Recent national and international reports indicate that a large proportion of adolescents exceed recommended daily screen-time limits and engage with multiple platforms concurrently, raising concerns about potential impacts on sleep, physical activity, and mental health [3]. At the same time, digital environments also provide opportunities for social connection, health information seeking, and access to mental health resources, underscoring the need to understand both the potential benefits and harms of digital engagement for youth well-being [4]

A growing body of empirical research has examined associations between social media use and adolescent mental health, with mixed but increasingly nuanced findings [5]. Several recent studies suggest that intensive or heavy use of social media is associated with higher levels of depressive symptoms, anxiety, and poor sleep, particularly among girls and young people with pre-existing vulnerabilities, although effect sizes are generally small to moderate [6,7]. Other work highlights that online social support, positive interactions, and access to digital mental health resources can mitigate some risks and, in some cases, promote improved well-being, illustrating the complex and bidirectional nature of these relationships [8,9].

Beyond social media, concerns about problematic or addictive patterns of digital technology use have intensified, with recent systematic reviews linking digital addiction, including problematic internet use, smartphone overuse, and gaming disorder, to poorer mental health, academic difficulties, and impaired social functioning in young people [10]. Emerging evidence also associates high levels of screen time and sedentary digital behaviors with higher risk of obesity, reduced physical activity, and disrupted sleep, although findings vary by age, sex, and type of digital activity [11]. At the same time, digital health technologies and serious games have shown promise for promoting physical activity, cognitive skills, and emotional regulation, suggesting that the health impacts of digital technologies depend on how, why, and in what contexts they are used [11].

### Evidence Gaps and the Need for Longitudinal Research

Despite rapid growth in the literature, the current body of evidence is dominated by studies that are cross-sectional, focus on single platforms or outcomes, or are conducted in high-income countries, limiting understanding of causal pathways, developmental trajectories, and contextual factors such as socioeconomic position and digital literacy. Recent protocols and initiatives have called for more comprehensive approaches that examine diverse forms of digital technology use, multiple health domains, and inequities in access and risks, highlighting the need for syntheses that map this emerging evidence base [11]. Several reviews and expert statements emphasize that strong longitudinal evidence on the long-term health consequences of digital engagement in young people remains limited, particularly in low- and middle-income settings. In 2021, a joint commission of *The Lancet* and *Financial Times* called for more longitudinal studies to be conducted with young people, particularly in low- and middle-income countries (LMICs) to understand the impact of different digital determinants of young people’s health [12]. Subsequent expert work has echoed these concerns, noting that current studies are often short term, rely heavily on self-reported screen use, and provide only fragmented evidence on mechanisms. Consequently, new longitudinal research agendas have been recommended [11,13]. Researchers, policymakers, and educators continue to grapple with understanding the effects of digital technology use on youth health, development, and education [13]. Longitudinal studies like Young Lives provide critical insights into how digital access, skills, and youth outcomes evolve over time, particularly in LMICs. Their findings underscore the importance of digital literacy and equitable access, highlighting how digital inclusion—or lack thereof—affects health and educational disparities [14]. Similarly, the Global Kids Online initiative explores how children’s digital engagement influences their social, emotional, and cognitive development, emphasizing how unequal access to technology can deepen existing inequalities [15].

The Health Behavior in School-Aged Children (HBSC) study has also been instrumental in tracking the impact of digital technologies on youth well-being. By examining digital device usage and its correlation with mental and physical health, HBSC data reveal growing concerns over social media’s influence on mental health, particularly in the wake of COVID-19, when digital communication became a crucial social lifeline [16]. These studies collectively illustrate the multifaceted relationship between digital engagement and youth outcomes, reinforcing the need for continued research to address the evolving challenges and opportunities presented by digital technologies.

While long-term research projects such as Young Lives, Global Kids Online, and HBSC offer important insights into how young people’s use of digital technologies is evolving, significant evidence gaps exist regarding the long-term effects of digital technology use on different aspects of young people’s health and well-being.

### Objectives of This Scoping Review

The purpose of scoping reviews is to synthesize evidence and assess the scope of existing literature on a topic. The objective of this scoping review is to systematically identify and map the breadth of longitudinal evidence on the relationships between everyday digital technology use and the health and well-being of young people under the age of 25 years. For the purposes of this review, we define longitudinal studies as empirical studies that collected data from the same participants at 2 or more time points separated by at least 3 months, allowing examination of changes in digital technology exposure and health outcomes over time. Specifically, the review seeks to inform future research directions by (1) charting the characteristics of existing longitudinal studies (including populations, types of digital technologies, outcomes, and settings), (2) summarizing how these studies conceptualize and measure digital engagement and health outcomes, and (3) identifying gaps and priorities for future longitudinal research.

Conceptually, this review is informed by emerging work on digital determinants of health, which examines how the various characteristics, features, uses, and governance of digital technologies and digital ecosystems influence health-related behaviors, health outcomes, and health care delivery [17]. For example, platform design, accessibility, data practices, and digital literacy have been identified as interacting with traditional social determinants to produce unequal risks and benefits of digital technologies across youth populations [18,19]. In addition, we draw on Bronfenbrenner ecological systems theory, which views young people’s development as shaped by interacting influences at multiple levels, from family and peers to schools, communities, and broader sociotechnical and policy environments [20]. Everyday digital engagement can be understood as a proximal process embedded within these nested systems, with bidirectional influences whereby youth both shape and are shaped by their use of digital technologies.

## Methods

### Search Strategy

This scoping review was conducted and reported in accordance with the PRISMA-ScR (Preferred Reporting Items for Systematic Reviews and Meta-Analyses Extension for Scoping Reviews) [21]. A comprehensive literature search was conducted in PubMed, Embase, and PsycArticles to identify relevant studies published between 2014 and 2024. Search strategies were developed using the PICOTS (Patient Population, Intervention, Comparator, Outcomes, Timing, Setting) framework (Table 1). These 3 databases were chosen because they collectively cover a wide range of biomedical, psychological, and public health research on digital technologies and youth health and were fully accessible within our institutional subscriptions. Keywords related to the specified population and technologies were used to refine searches across databases. Boolean operators were employed to combine terms such as “youth,” “adolescent,” “digital technology,” and “longitudinal study” to target relevant research. Database-specific search strings for PubMed, Embase, and PsycArticles, including all search terms, field tags, and limits, are provided in Multimedia Appendix 1.

**Table 1.** PICOTS (patient population, intervention, comparator, outcomes, timing, setting) framework used to structure the search strategy for this scoping review of longitudinal studies examining associations between everyday digital technology use and health and well-being outcomes among young people aged 25 years or younger (2014‐2024).

Question	Keywords
P (patient or population)	Youth, adolescent, child, childhood
I (intervention)	Digital technology, social media, video games, internet use
C (comparator)	Not applicable for this study
O (outcomes)	No keywords applied to enable broad inclusion of different health and well-being outcomes
T (timing)	2014‐2024
S (study type)	Longitudinal

The reporting of the search strategy follows key elements of the PRISMA-ScR checklist (Checklist 1). Search results from all databases were imported into Rayyan for reference management; Rayyan’s deduplication function was used to identify duplicate records, which were then reviewed and confirmed by the first author before screening [22].

Given the exploratory nature of this scoping review, formal preregistration was not performed; however, a documented protocol was established and shared to enhance rigor and transparency. Quality appraisal was not conducted as the objective of the review was to map the literature and not assess quality of evidence. For similar reasons, no attempt was made to estimate pooled effect sizes or formally assess risk of bias.

Search strategies were developed using the PICOTS framework as a guide and tailored to each database, combining controlled vocabulary (eg, MeSH, Emtree) and free-text terms for youth (child, adolescent, young adult), digital technologies (digital technology, internet use, social media, video games, digital health, mobile apps, telemedicine, wearable electronic devices), and longitudinal study designs [23, 24]. Detailed search strings for each database and aim are provided in Multimedia Appendix 1 to enable replication.

### Selection of Sources of Evidence

A total of 456 studies were initially identified in the screening process. After applying the inclusion and exclusion criteria detailed in Table 2, 267 studies were deemed relevant to the research question, focusing on longitudinal studies that examine the impact of digital technologies on youth well-being. The remaining 189 studies were excluded due to irrelevance, as they did not meet the criteria for exploring the relationship between everyday digital technology use and youth health outcomes in a longitudinal framework. This rigorous screening process ensured that only studies directly aligned with the research objectives were included for synthesis and analysis.

**Table 2.** Inclusion and exclusion criteria applied during screening of longitudinal empirical studies examining associations between everyday digital technology use and health or well-being outcomes among children, adolescents, and young adults (≤25 y) published between 2014 and 2024.

Category	Inclusion criteria	Exclusion criteria
Article type	Longitudinal empirical studies (cohort, panel, or repeated-measures designs) examining associations between everyday digital technology use and health or well-being outcomes in youth	Cross-sectional studies, case reports, qualitative-only studies, reviews, commentaries, editorials, conference abstracts without full data, and nonempirical papers
Population	Children, adolescents, and young adults aged ≤25 years (or samples with a mean age ≤25, SD 5 y) from any country or setting	Studies focusing exclusively on adults >25 years or mixed-age samples where data for participants ≤25 years cannot be disaggregated
Digital technology exposure	Everyday digital technologies such as internet use, social media, online gaming, smartphones, and other general digital media exposures	Studies focusing solely on highly specialized clinical technologies (eg, neurosurgical devices) or nondigital interventions; technology not related to personal digital media use
Outcomes	Health and well-being outcomes, including mental, physical, behavioral, social, or cognitive outcomes assessed at follow-up	Studies without health or well-being outcomes (eg, usability only) or outcomes unrelated to youth health (eg, purely technical performance metrics)
Study design and timeframe	Longitudinal design with at least 2 time points; published between 2014 and 2024 in peer-reviewed journals	Single time point (cross-sectional) designs; studies outside the 2014‐2024 window or not peer reviewed
Language	Articles published in English	Articles published in languages other than English

Titles and abstracts were screened by the first author using predefined inclusion and exclusion criteria developed together with the second author. Any uncertainties were discussed with the second author before making final eligibility decisions. Full texts of potentially eligible articles were then assessed against the same criteria.

### Data Extraction

Data from all included studies were extracted by the first author, with methodological guidance and spot checks provided by the second author to ensure accuracy and consistency. The form was piloted on a sample of studies and refined prior to full data charting. Data charting was conducted using a standardized Microsoft Excel form developed specifically for this review. The first author initially charted data from a small sample of eligible studies to pilot the form, after which the structure and definitions of each field were refined in consultation with the second author to improve clarity and consistency. The first author then charted data from all included studies, and the second author performed targeted checks of randomly selected entries to verify accuracy and resolve any uncertainties.

For each study, we extracted key data to organize and analyze the findings effectively. The following variables were recorded:

1. *Title:* The full title of the study for accurate identification
2. *Abstract:* A summary outlining the study’s aims, methods, and results
3. *Journal:* The journal in which the study was published
4. *Volume, issue, pages:* Journal volume, issue number, and page range for citation purposes
5. *Authors:* The research team behind the study
6. *DOI:* The Digital Object Identifier (DOI) for easy access to study
7. *Theme:* The main research focus (eg, social media, video games, mobile applications)
8. *Subtheme:* A more specific focus within the theme (eg, for social media—“Impact on mental health”)
9. *Age group:* The age category of the participants (eg, child, adolescent, young adult)
10. *Study type:* The design of the study (eg, longitudinal, cross-sectional)
11. *Outcome type:* The health or well-being outcome being assessed (eg, mental health, cognitive development)
12. *Gender:* The gender breakdown of the study population (eg, male, female, mixed)
13. *Geographical focus:* The region or country where the study took place
14. *Study name:* The official name of the study, if available
15. *Leading institution:* The institution responsible for the research

### Themes Identified

Data charted from individual studies were synthesized using an inductive-deductive thematic approach appropriate for scoping reviews. Study aims and prior conceptual work informed an initial set of themes, which was then refined as new themes and insights emerged from the data. After familiarization with the charted data, the first author grouped studies according to similarities in digital technology type, health or well-being outcomes, and study aims. These preliminary groupings and definitions were then reviewed with the second author, who suggested refinements until consensus was reached on 6 overarching themes. Each included study was assigned to 1 primary theme based on its main focus; when a study spanned multiple topics, the dominant exposure-outcome relationship was used to guide classification. Within each theme, findings were synthesized descriptively, focusing on patterns in study designs, measures of digital engagement and outcomes, direction and consistency of reported associations, and notable gaps in populations or contexts. No meta-analysis or quantitative pooling of effect estimates was performed, in line with the objectives of a scoping review.

The following 6 key themes were assigned to the studies based on their content and focus:

1. *Social media use and mental health:* Studies exploring the relationship between social media engagement and various mental health outcomes, including anxiety, depression, and social well-being
2. *Digital addiction and behavioral outcomes*: Research investigating the addictive potential of digital technologies and their effects on behavior, such as compulsive use of social media or video games
3. *Physical activity and digital technology:* Studies examining the interaction between physical activity levels and digital technology use, including how digital technologies may influence exercise behaviors
4. *Digital health technologies and cognitive development:* Research focused on the use of digital tools, such as mobile apps and telemedicine, to enhance cognitive development and support educational outcomes in young people
5. *Parental influence and digital technology:* Studies exploring the role of parents in shaping their children’s use of digital technologies, including the impact of parental guidance on digital behaviors
6. *Digital well-being and risk behaviors:* Research addressing how digital technology use impacts risk behaviors such as substance abuse, risky sexual behavior, and online safety concerns

To facilitate reproducibility, the full search strategies, screening decision rules, and data-extraction template are available in Multimedia Appendix 1 and from the corresponding author upon reasonable request. The completed PRISMA-ScR checklist is provided in Checklist 1.

## Results

### Study Selection and Overview of Included Studies

**Figure 1.**
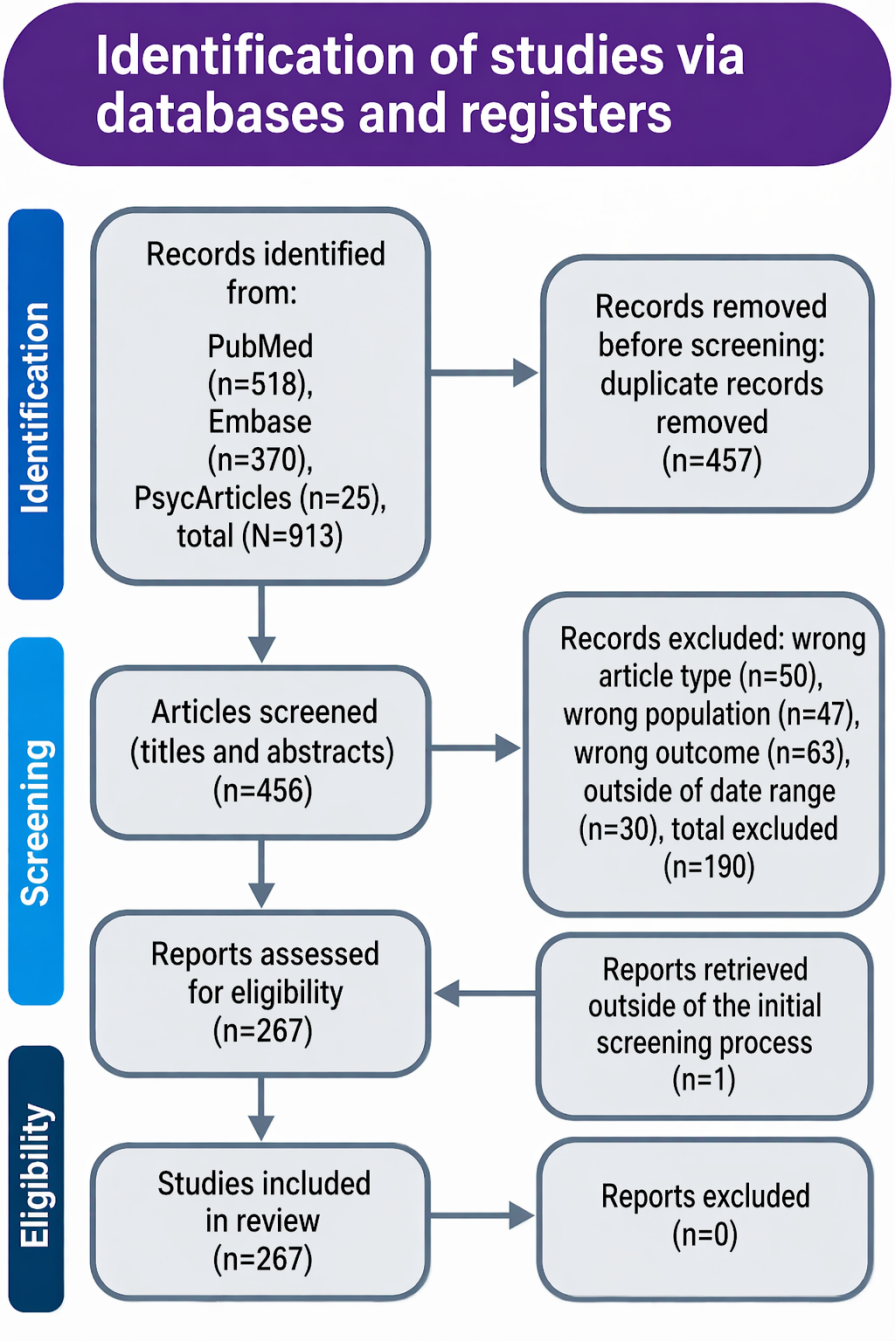
PRISMA-ScR (Preferred Reporting Items for Systematic Reviews and Meta-Analyses Extension for Scoping Reviews) flow diagram illustrating identification, screening, eligibility assessment, and inclusion of longitudinal studies examining everyday digital technology use and health or well-being outcomes among young people aged 25 years or younger (2014‐2024).

The scoping review encompassed a total of 267 studies, which provided insight into various aspects of digital technology use and its impact on the health and well-being of young people over time. Internet use (n=201 studies), social media (n=140 studies), and gaming (n=83 studies) dominated the themes. Mental health was the most frequently assessed outcome, with a focus on anxiety and depression. Figure 1 presents the PRISMA-ScR flow of records through identification, screening, eligibility assessment, and inclusion. Across databases, 913 records were retrieved (PubMed n=518, Embase n=370, and PsycArticles n=25), of which 457 duplicates were removed before screening, leaving 456 unique records for title and abstract review. Of these, 190 records were excluded (wrong article type n=50, wrong population n=47, wrong outcome n=63, outside of date range n=30), with 266 studies remaining. One additional report was identified and retreived outside the initial screening process, resulting in 267 studies being included in the review.

**Figure 2.**
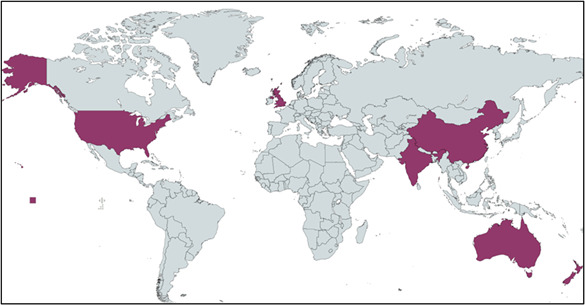
Geographic distribution of the 267 longitudinal studies included in the scoping review examining digital technology use and youth health outcomes, highlighting the predominance of studies conducted in high-income countries.

Geographically, 15% (40/267) of studies originated from LMICs, with the majority coming from high-income settings such as the United States (n=76 studies) and Australia (n=15 studies; Figure 2). Nearly half (131/267, 49%) were published post 2020, reflecting heightened interest during the COVID-19 pandemic.

The analysis of these longitudinal studies revealed 6 key themes: social media use and mental health; digital addiction and behavioral outcomes; physical activity and digital technology; digital health technologies and cognitive development; parental influence and digital technology; and digital well-being and risk factors. Each theme highlights important findings related to the impact of digital technologies on health outcomes for youth. Table 3 summarizes the number of included studies by thematic category, country income group, and predominant age group, illustrating the concentration of research in high-income settings and among adolescents and young adults.

**Table 3.** Distribution of 267 longitudinal studies included in the scoping review examining digital technology use and youth health outcomes, categorized by thematic focus, country income group, and predominant participant age group.

Theme	Studies (N=267), n	Main country income groups^a^	Predominant age groups^b^
Social media use and mental health	69	Mostly high-income; some upper-middle-income	Adolescents, young adults
Digital addiction and behavioral outcomes	102	High-income and upper-middle-income	Adolescents, young adults
Physical activity and digital technology	18	Mostly high-income	Children, adolescents
Digital health technologies and cognitive development	33	Mostly high-income	Children, adolescents
Parental influence and digital technology	11	Mostly high-income	Children, adolescents
Digital well-being and risk behaviors	34	High-income and upper-middle-income	Adolescents, young adults

a Income groups classified according to the World Bank categories reported for study countries.

b Age groups as charted during data extraction: children (<12 y), adolescents (12‐17 y), and young adults (18‐25 y).

**Figure 3.**
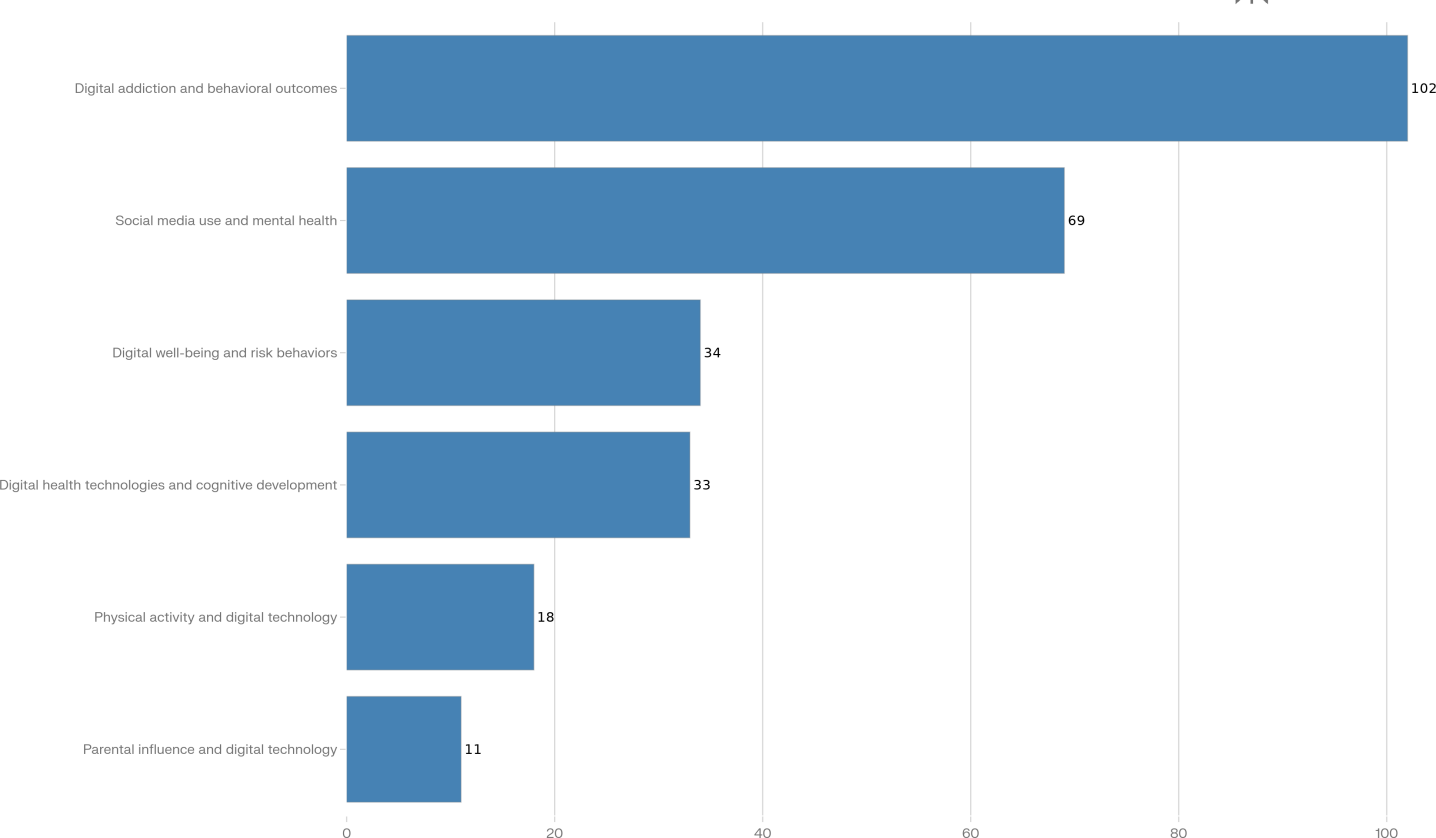
Number of longitudinal studies included in the scoping review by thematic category of digital technology use and youth health outcomes.

The distribution of longitudinal studies across the 6 thematic categories is presented in Figure 3. This visualization highlights the predominance of research on digital addiction and mental health outcomes compared to a relative scarcity of longitudinal studies on physical health and cognitive development impacts.

### Theme 1: Social Media Use and Mental Health

A total of 69 longitudinal studies focused on the effects of social media on adolescent mental health. Of these, 45 studies examined depression and anxiety, showing a strong association between increased social media use and negative mental health outcomes, particularly depression and anxiety. Six studies explored the influence of social media on self-esteem and body image, indicating that social media can exacerbate body dissatisfaction, especially among adolescent girls [25-30]. Nine studies addressed the issue of cyberbullying, which was found to significantly affect mental health, with higher risks of depression, anxiety, and suicidal ideation [31-39]. Nine studies also highlighted positive aspects of social media use, such as online support groups and mental health resources [34,40-48]. Overall, the studies suggest that social media use has a complex, bidirectional relationship with mental health, with both positive and negative outcomes.

These studies reveal an important distinction between intensive and problematic social media use among adolescents. Intensive use, defined as high frequency or duration of engagement, was not consistently associated with adverse mental health outcomes, with several studies reporting no significant correlation between frequency of social media use and measures such as depression, anxiety, or stress [16,49]. In contrast, problematic social media use, characterized by addiction-like symptoms such as compulsive engagement, withdrawal, and loss of control, was moderately and consistently linked to increased symptoms of depression, anxiety, stress, and loneliness [16,50]. For example, meta-analytic and longitudinal studies reported that increases in problematic social media use are significantly associated with heightened depressive symptoms and loneliness over time, while intensive use alone does not show these associations [49,50]. These findings underscored the importance of differentiating between patterns of social media engagement, as problematic use appeared to pose a greater risk to adolescent mental health than high-frequency use alone.

Of the 69 studies in this theme, roughly two-thirds reported predominantly adverse associations between problematic or intensive social media use and later mental health outcomes, around one-quarter reported mixed or null findings, and a small minority emphasized mainly positive or buffering effects of online engagement. Taken together, the longitudinal evidence suggested that how adolescents use social media matters more than how much they use it, with addiction-like, emotionally dysregulated engagement aligning more closely with stress-generation and differential-susceptibility models than simple “screen-time” explanations. This theme highlighted the need for future studies to explicitly test mechanisms such as social comparison, sleep disruption, and online victimization as pathways linking problematic use to later depressive and anxiety symptoms.

### Theme 2: Digital Addiction and Behavioral Outcomes

This theme addressed longitudinal studies of addictive-like behaviors fostered by everyday technology use (eg, internet, gaming, smartphone addiction) and their associations with mental health and behavioral functioning.

This review incorporated 102 studies on the impact of digital addiction. The majority of the studies (n=56 observational) demonstrated a link between excessive or compulsive digital technology use, particularly internet and gaming addiction, and behavioral issues like anxiety, depression, and poor social functioning. Notably, internet addiction (n=39 studies) and gaming addiction (n=38 studies) were strongly correlated with poor mental health and academic performance, while smartphone dependency (n=4 studies) and behavioral disorders (n=20 studies) further exacerbated existing mental health issues [51-57]. Among 36 intervention studies, 54% (144/267) reported positive outcomes in terms of digital addiction treatment and management. However, a significant portion of studies revealed harmful effects of excessive digital use, which included disrupted sleep, increased mental health symptoms, and social isolation. Longitudinal research emphasized the long-term psychological consequences, such as increased depression and anxiety, associated with digital addiction [58-60].

Across the 102 digital addiction studies, most observational studies found that higher levels of internet or gaming addiction predicted worsening mood or functioning over time, whereas only a minority of intervention trials demonstrated sustained reductions in both addictive use and mental health symptoms. Overall, findings in this theme support conceptualizing digital addiction as part of a broader cluster of self-regulation and impulse-control difficulties, rather than as an isolated problem of device use. Several studies and reviews also highlighted how specific design features of social media platforms and games, such as variable and unpredictable rewards and always-on social notifications, were intended to maximize engagement and could reinforce compulsive checking and prolonged use, particularly among adolescents [61,62]. Longitudinal patterns linking persistent addiction-like use with worsening mood, school functioning, and social withdrawal pointed to the importance of interventions that targeted underlying regulatory skills and family or school contexts alongside usage reduction.

### Theme 3: Physical Activity and Digital Technology

A total of 18 longitudinal studies were analyzed under this theme. A total of 13 studies explored the relationship between sedentary screen time and reduced physical activity. Two studies researched the effectiveness of digital tools, including fitness apps and activity trackers, in increasing physical activity levels. A further 3 studies focused on digital gamification or exergames designed to increase movement. Studies on sedentary screen time pointed to the negative effects of excessive screen time, which were associated with physically inactive lifestyles and an increased risk of obesity, particularly among girls. A bidirectional relationship was observed, with excessive screen time negatively impacting physical activity, while physical inactivity also exacerbated screen time use [63,64]. Only a small subset of intervention and gamification studies found sustained increases in physical activity linked to digital tools.

Across studies, digital technologies emerged as a double-edged tool for youth physical health: design features that scaffolded motivation and feedback could nudge activity, while unstructured screen use reinforced sedentary routines and obesogenic environments. These findings underscored the value of moving beyond total “screen time” metrics toward frameworks that differentiate health-promoting, deliberately designed movement-supporting tools from passive, displacement-oriented digital use. Future research should focus on long-term interventions to increase physical activity and reduce sedentary behavior across diverse youth populations.

### Theme 4: Digital Health Technologies and Cognitive Development

A total of 33 longitudinal studies focused on the relationship between digital technology use and cognitive development. Fifteen studies evaluated deliberately designed digital health or educational tools to support cognitive outcomes (mobile health or training apps, n=10; telemedicine or telehealth for cognitive disorders, n=2; e-learning or serious game platforms, n=3), while 18 studies examined everyday digital media use (eg, overall screen time, general device or internet use) in relation to cognitive development. These studies generally indicated positive effects of digital health technologies on cognitive and behavioral outcomes, including attention improvement and executive function through exergames and mobile applications [65-76]. However, concerns regarding the long-term effectiveness of these technologies were prevalent, with many showing only short-term improvements or mixed results in sustained behavior change.

Eighteen studies explored the impact of everyday digital technology use on cognitive development. Across these studies of everyday digital technology use, higher overall screen time and background television exposure were frequently associated with weaker performance or slower gains in language, executive function, or school readiness, especially in younger children, although effect sizes were generally small. These patterns reinforced concerns about heavy, nonstructured screen use and support calls for more balanced, developmentally appropriate digital engagement

Of the 33 studies in this theme, around two-thirds documented short-term improvements in at least 1 cognitive or learning outcome following exposure to digital health or educational technologies (eg, training apps, exergames, e-learning platforms), whereas approximately one-third reported mixed, null, or unsustained effects, particularly at longer follow-up or in relation to more diffuse measures of everyday screen use.

Evidence in this theme suggested that digital health technologies are promising but not yet consistently transformative tools for youth cognitive development, with many benefits appearing short-term or context-dependent. Conceptually, this suggests that technology functions as an amplifier of existing learning environments and supports, rather than a stand-alone solution, and that sustained gains likely require integration with offline pedagogy, family engagement, and broader educational resources. At the same time, findings from observational studies in this theme underscored the importance of actively mitigating potential harms from heavy, nonstructured or background screen exposure, through limits on overall screen time, attention to content quality, and alignment of digital use with age-appropriate, offline learning and caregiving routines

### Theme 5: Parental Influence and Digital Technology

A total of 11 longitudinal studies analyzed parental influence on children’s digital behaviors and online safety. The studies revealed that parental monitoring and control of screen time positively influenced youth behavior, with several studies showing improved outcomes in mental health and behavioral issues. However, some studies highlighted the challenges of treatment adherence and the need for refinement in interventions aimed at parental involvement. Parental education about digital literacy and online safety was also shown to have a beneficial impact, although the effects were small and varied depending on the type of screen activity [76-80]. Future research should refine interventions to better support family dynamics and address treatment attrition.

Among the 11 studies examining parental influence, most found that some form of active monitoring, co-use, or guidance was linked to modest improvements in mental health, behavior, or online safety indicators, whereas purely restrictive strategies showed smaller and less consistent benefits and occasionally coincided with more conflictual digital use.

Collectively, these studies reinforce models of “co-regulation,” in which parents shape not only how much time children spend online but also the meanings and risks attached to that time. Modest but consistent benefits of warm, structured guidance, compared with either lax or highly controlling approaches, highlighted the need for interventions that bolster parents’ digital literacy and relational engagement rather than focusing solely on restrictive screen-time limits.

### Theme 6: Digital Risk Factors

This theme focused on digital risk behaviors (eg, cyberbullying, sexting, exposure to harmful content) and how they co-occurred with and predict substance use, aggression, and other offline risks. This theme covered 34 longitudinal studies on risky digital behaviors—such as cyberbullying, sexting, and exposure to harmful content—and their relationship with other risk factors that influence young people’s well-being, including substance abuse and aggressive behavior. Studies indicated that increased social media exposure and engagement in online activities such as cyberbullying, sexting, and online grooming correlated with higher risks of substance abuse and aggressive behaviors [30,81]. Furthermore, substance abuse was found to be linked to digital media use, particularly in adolescents, with frequent social media users reporting higher rates of alcohol, tobacco, and drug use compared to their peers [82-95]. The studies underlined the need for better regulation and monitoring of digital content to mitigate these risks [96]. Studies suggested that early media exposure could influence long-term risky behaviors, and intervention strategies focusing on media literacy and parental monitoring were highlighted as effective approaches [97-99].

Findings in this theme suggested that risky online behaviors often co-occurred with broader clusters of offline vulnerability, supporting syndemic and developmental-cascade perspectives in which digital and offline risks reinforced one another over time. Interventions that combine media literacy, skills for navigating online social pressures, and targeted support for substance use and aggression appeared more theoretically justified than approaches that address digital and offline risks in isolation.

Overall, the findings from these 6 themes underscored the complexity of digital technology’s influence on youth health, with both positive and negative outcomes depending on usage patterns and the context of engagement. Across all 6 themes, the evidence depicted everyday digital technology use as a contextual, socially embedded determinant of youth health, with risks and benefits shaped by prior vulnerabilities, family and school environments, and the specific functions that digital tools serve in young people’s lives.

## Discussion

### Principal Findings

The aim of this scoping review was to systematically identify and map the breadth of longitudinal evidence on the relationships between everyday digital technology use and the health and well-being of young people under the age of 25 years. The review sought to (1) chart the characteristics of existing longitudinal studies, (2) summarize how these studies conceptualize and measure digital engagement and health outcomes, and (3) identify gaps and priorities for future longitudinal research.

The review found that most longitudinal studies published between 2014 and 2024 focused on 3 aspects of technology use: internet browsing (n=201), social media (n=140), and gaming (n=83). Digital addiction and mental health, particularly anxiety and depression, were the dominant health outcomes studied. A major gap in the geographical scope of studies was identified: only 15% (40/267) of the included studies originated from LMICs.

### Conceptual Implications

Taken together, the 6 dominant themes identified within the longitudinal studies support an ecological view of digital engagement and youth health, in which associations are rarely linear or uniform but instead depend on who is using which technologies, in what social and structural contexts, and for what purposes. For example, problematic, dysregulated use in the context of family conflict and offline adversity appears more strongly linked to subsequent internalizing symptoms and risky behaviors than similar levels of use in supportive environments, consistent with ecological and digital-determinants perspectives. Social media use was linked to increased loneliness and distress, especially during the COVID-19 pandemic. At the same time, positive uses, such as accessing health information or supportive communities, illustrate how digital spaces can also function as protective resources when embedded within enabling microsystems and macrosystems [18,20].

By combining thematic synthesis with simple frequency counts across technologies, outcomes, age groups, and regions, this review functions as an evidence map that highlights both densely studied areas (eg, social media and internalizing symptoms) and important gaps (eg, physical health, cognitive development outcomes, and research in low- and middle-income settings).

### Cross-Cutting Patterns

Across themes, several cross-cutting patterns emerge from the longitudinal evidence. First, associations between digital engagement and youth health are generally small in magnitude, often contingent on baseline vulnerabilities, offline context, and the quality rather than the quantity of engagement, which aligns with differential susceptibility and person-by-context interaction models. Second, studies that distinguish between specific modes of use (eg, passive vs active social media use, recreational vs problematic gaming) more consistently report directional relationships than those that rely on global screen-time measures, suggesting that conceptual models focused on “time displacement” alone are insufficient. Third, very few longitudinal studies explicitly test mediating pathways (eg, sleep disruption, social comparison, cyberbullying), limiting the ability to draw firm causal conclusions even when temporal ordering is clear. Digital addiction and digital risk behaviors represent related but distinct pathways through which online engagement influences youth health; these themes are therefore discussed separately but cross-referenced where studies span both domains.

### Implications for Theory and Methods

Taken together, the mapped evidence supports a shift away from simple “screen-time” narratives toward frameworks that emphasize conditional and pathway-specific effects of digital engagement on youth health, for example, models in which digital experiences amplify existing mental health risks for some young people while offering compensatory social support or health information for others. Longitudinal designs that incorporate repeated measures of context, mediators, and offline supports are needed to move from description toward testing such conceptual models.

### Implications for Public Health and Policy

From a public health perspective, these findings support a shift from individual responsibility narratives toward policies that address the digital environments in which young people spend time [100]. First, educators and health systems can use this evidence to develop age-appropriate digital literacy and mental health literacy programs that help young people recognize harmful content, manage problematic use, and leverage supportive online spaces, particularly for those already facing offline vulnerabilities [101]. Second, the concentration of harms around specific platform features, such as algorithmic amplification of extreme content and design elements that encourage compulsive engagement, underscores the need for regulation that embeds “safety-by-design” principles, transparency, and accountability for industry actors [102]. Third, inequities in who benefits from digital health technologies point to the importance of treating digital connectivity, skills, and protections as digital determinants of health and of integrating these into broader strategies to reduce mental health and well-being disparities among young people [103].

Our analysis reveals a marked increase in publications addressing digital technology and youth health since 2020. Most studies focus on mental health, particularly the effects of social media use, while fewer address physical health, educational outcomes, or equity considerations. Notably, there is a paucity of research from LMICs and limited longitudinal evidence on the long-term impacts of digital technology use. Given that 15% (40/267) of the included studies originated from LMICs, our conclusions about global patterns of digital engagement and health should be interpreted cautiously and viewed as more representative of high-income settings. Expanding research in these settings is crucial to understanding disparities in access and digital literacy. These gaps underscore the need for future research in underrepresented areas and populations. Additionally, the diversity of study designs reflects the evolving nature of this field, with a trend toward more sophisticated methodologies in recent years.

### Strengths and Limitations

This review has several strengths, including the systematic mapping of longitudinal evidence across multiple domains of digital technology use and youth health outcomes. This scoping review synthesizes available longitudinal evidence on everyday digital technology use and youth well-being, highlighting predominant research foci and key gaps rather than providing a definitive assessment of effects. The findings point to priorities for future longitudinal research and for the careful design and evaluation of digital policies and interventions, particularly in underrepresented regions and populations.

This scoping review has several limitations that should be considered when interpreting the findings. First, the search was restricted to 3 databases (PubMed, Embase, and PsycArticles), to articles published in English, and to studies from 2014 to 2024, which means that relevant longitudinal research indexed elsewhere, published in other languages, or outside this time window may have been missed. The search was limited to PubMed, Embase, and PsycArticles; other multidisciplinary databases such as Scopus or Web of Science were not searched, which means that some relevant longitudinal studies indexed elsewhere may have been omitted.

Second, consistent with the aims and methodology of a scoping review, we did not conduct a formal meta-analysis, and the synthesis is descriptive rather than quantitative; therefore, the review cannot determine the direction or magnitude of causal effects. Third, most included studies were conducted in high-income countries, so the patterns summarized here may not be generalizable to low- and middle-income settings or to underrepresented youth populations.

Although a formal quality assessment or risk-of-bias appraisal was not undertaken in line with scoping review guidance, several recurrent methodological limitations should be noted at the level of the primary studies. Many relied on self-reported digital use and health outcomes, which are vulnerable to recall and social desirability biases, and follow-up periods were often relatively short, limiting inferences about long-term trajectories. Residual confounding is also likely, as few studies used repeated measures, time-varying covariates, or strong causal designs. Additionally, most samples were drawn from high-income countries and school- or clinic-based populations, which constrain generalizability. Taken together, these patterns suggest that both harmful and beneficial associations between digital engagement and youth health may be misestimated in the existing longitudinal evidence base and point to the need for more rigorous and diverse designs in future work.

### Directions for Future Research

The findings from this study reinforce the call from the *Lancet* and *Financial Times* Commission for more longitudinal research on the digital determinants of young people’s health and well-being. Future longitudinal research should examine the long-term effects of everyday technology use and other forms of digital engagement on different dimensions of youth physical health, mental health, and well-being, including emerging technologies like virtual reality and artificial intelligence. Important objectives of these studies should be to increase our collective understanding of which features of digital technologies and platforms are most likely to promote or undermine health outcomes and why some young people are more vulnerable to the negative impacts of digital technology use than others. Greater effort is needed to generate evidence from LMICs to understand how sociocultural and economic contexts shape the impacts of technology use. Additionally, studies should examine the role of digital literacy in mitigating risks and tailoring interventions to diverse populations. Exploring digital interventions—such as literacy programs, online support communities, and mental health resources—will be essential in maximizing benefits while minimizing risks, ultimately fostering youth resilience and well-being.

### Conclusions

This scoping review highlights the rapid expansion of longitudinal research examining digital technology use and youth health, while also revealing important conceptual and geographical gaps in the evidence base. Most existing studies focus on mental health outcomes in high-income settings, with far fewer investigating physical health, cognitive development, or youth populations in LMICs. Future research should prioritize more diverse populations, stronger longitudinal designs, and clearer conceptual models to better understand how different forms of digital engagement shape young people’s health and well-being over time. Strengthening this evidence base will be essential for informing policies and interventions that maximize the benefits of digital technologies while mitigating potential harms.

## Supplementary material

10.2196/85094 Multimedia Appendix 1 Search strategies.

10.2196/85094 Checklist 1 PRISMA checklist.

## Abbreviations

HBSC: Health Behavior in School-aged Children
LMICs: low- and middle-income countries
PRISMA-ScR: Preferred Reporting Items for Systematic Reviews and Meta-Analyses Extension for Scoping Reviews
