# Supplementary material for: Everyday Digital Technology Use and Youth Health: Scoping Review of Longitudinal Studies
Source: JMIR Public Health Surveill. 2026 Apr 27;12:e85094. doi: 10.2196/85094 (PMC13120553; doi:10.2196/85094)
Supplement: Multimedia Appendix 1 [file publichealth-v12-e85094-s001.docx]

# **Appendix 1**

**Full database search strategies**

This appendix presents the complete search strategies for each database used in the scoping review. Searches were structured using PICOTS elements (Population, Intervention/Exposure, Comparator, Outcomes, Timing, Study design) and tailored to each database. Limits were applied to identify peer-reviewed longitudinal studies involving youth (≤25 years) and examining everyday digital technologies or digital health technologies between 2014 and 2024.

**A1. PubMed**

**Population terms**

- Young Adult[Mesh] OR Child[Mesh] OR Adolescent[Mesh] OR young[tiab] OR youth[tiab] OR adolescent*[tiab] OR adolescen*[tiab] OR teen*[tiab] OR child*[tiab]

**Exposure terms**

- Digital Technology[Mesh] OR Internet[Mesh] OR Social Media[Mesh] OR Video Games[Mesh] OR digital technolog*[tiab] OR internet[tiab] OR online[tiab] OR social media[tiab] OR social network*[tiab] OR video game*[tiab] OR gaming[tiab] OR TikTok[tiab] OR Instagram[tiab] OR Facebook[tiab] OR YouTube[tiab]

**Study design terms**

- Longitudinal Studies[Mesh] OR longitudinal[tiab] OR “prospective study”[tiab] OR “panel study”[tiab] OR “cohort study”[tiab]

**Full PubMed search string**

text

(

Young Adult[Mesh] OR Child[Mesh] OR Adolescent[Mesh]

OR young[tiab] OR youth[tiab] OR adolescent*[tiab] OR adolescen*[tiab]

OR teen*[tiab] OR child*[tiab]

)

AND

(

Digital Technology[Mesh] OR Internet[Mesh] OR Social Media[Mesh] OR Video Games[Mesh]

OR digital technolog*[tiab] OR internet[tiab] OR online[tiab]

OR social media[tiab] OR social network*[tiab]

OR video game*[tiab] OR gaming[tiab]

OR TikTok[tiab] OR Instagram[tiab] OR Facebook[tiab] OR YouTube[tiab]

)

AND

(

Longitudinal Studies[Mesh] OR longitudinal[tiab]

OR "prospective study"[tiab] OR "panel study"[tiab] OR "cohort study"[tiab]

)

AND ("2014/01/01"[dp] : "2024/12/31"[dp])

**A2. Embase**

**Population**

- young adult*/exp OR young adult*:ti,ab OR child*/exp OR child*:ti,ab OR adolescent*/exp OR adolescent*:ti,ab OR childhood*:ti,ab OR teen*:ti,ab

**Exposure**

- internet use:ti,ab OR internet:ti,ab OR online:ti,ab OR "social media":ti,ab OR "social network*":ti,ab OR "video game*":ti,ab OR gaming:ti,ab OR "digital technolog*":ti,ab

**Study design**

- longitudinal study:ti,ab OR longitudinal:ti,ab OR cohort:ti,ab OR "prospective study":ti,ab OR "panel study":ti,ab

**Full Embase search**

text

(

'young adult'/exp OR 'young adult':ti,ab OR 'child'/exp OR child*:ti,ab

OR 'adolescent'/exp OR adolescent*:ti,ab OR childhood*:ti,ab OR teen*:ti,ab

)

AND

(

'internet use':ti,ab OR internet:ti,ab OR online:ti,ab

OR 'social media':ti,ab OR 'social network*':ti,ab

OR 'video game*':ti,ab OR gaming:ti,ab

OR 'digital technolog*':ti,ab

)

AND

(

'longitudinal study':ti,ab OR longitudinal:ti,ab OR cohort:ti,ab

OR 'prospective study':ti,ab OR 'panel study':ti,ab

)

AND [2014-2024]/py

**A3. PsycArticles**

text

AB(youth OR adolescent* OR adolescen* OR childhood OR child* OR teen*)

AND

TI(digital OR technology OR "social media" OR "social network*" OR "video game*" OR gaming OR internet OR online)

AND

TI("longitudinal study" OR longitudinal)

AND

DT(2014-2024)
